# Possible interactions between selected food processing and medications

**DOI:** 10.3389/fnut.2024.1380010

**Published:** 2024-04-12

**Authors:** Giuseppe Poli, Ettore Bologna, I. Sam Saguy

**Affiliations:** ^1^Department of Clinical and Biological Sciences, San Luigi Hospital, University of Turin, Turin, Italy; ^2^Medical Service Fondazione Piera Pietro and Giovanni Ferrero, Alba, Italy; ^3^The Robert H. Smith Faculty of Agriculture, Food & Environment, The Hebrew University of Jerusalem, Rehovot, Israel

**Keywords:** food processing, thermal processing, non-thermal processing, food-drug interaction, membrane transporters, cytochrome P450, drug bioavailability

## Abstract

The impact of food processing on drug absorption, metabolism, and subsequent pharmacological activity is a pressing yet insufficiently explored area of research. Overlooking food-processing-drug interactions can significantly disrupt optimal clinical patient management. The challenges extend beyond merely considering the type and timing of food ingestion as to drug uptake; the specific food processing methods applied play a pivotal role. This study delves into both selected thermal and non-thermal food processing techniques, investigating their potential interference with the established pharmacokinetics of medications. Within the realm of thermal processing, conventional methods like deep fat frying, grilling, or barbecuing not only reduce the enteric absorption of drugs but also may give rise to side-products such as acrylamide, aldehydes, oxysterols, and oxyphytosterols. When produced in elevated quantities, these compounds exhibit enterotoxic and pro-inflammatory effects, potentially impacting the metabolism of various medications. Of note, a variety of thermal processing is frequently adopted during the preparation of diverse traditional herbal medicines. Conversely, circumventing high heat through innovative approaches (e.g., high-pressure processing, pulsed electric fields, plasma technology), opens new avenues to improve food quality, efficiency, bioavailability, and sustainability. However, it is crucial to exercise caution to prevent the excessive uptake of active compounds in specific patient categories. The potential interactions between food processing methods and their consequences, whether beneficial or adverse, on drug interactions can pose health hazards in certain cases. Recognizing this knowledge gap underscores the urgency for intensified and targeted scientific inquiry into the multitude of conceivable interactions among food composition, processing methods, and pharmaceutical agents. A thorough investigation into the underlying mechanisms is imperative. The complexity of this field requires substantial scrutiny and collaborative efforts across diverse domains, including medicine, pharmacology, nutrition, food science, food technology, and food engineering.

## Introduction

The health effects of bioactive substances in the human body are affected by several factors, including food composition and processing conditions, storage conditions, light and heat, among others. These factors greatly limit the stability and bioavailability of bioactive substances (1).

Adverse food-drug interactions can be serious and, in extreme cases, may result in severe health consequences, including death. In children and older adults, undetected disadvantageous food-drug interactions may lead to serious morbidity and mortality and be misdiagnosed as chronic disease progression (2). For instance, recent recognition of the effects of certain foods on many drugs metabolized by CYP450 families or drugs susceptible to chelation and absorption have increased awareness for prevention of food-drug negative interactions (2–4).

However, the possible impact on health by the processes utilized in producing the various foodstuffs and their possible interactions with medications have not been adequately covered. A recent Google Scholar search (Dec. 4, 2023) yielded (for “effect of food on drug” and “effect of food processing on drug,” or medication) 606 and 0 hits, respectively. As new technologies and novel drugs emerge, interactions between food processing and drugs can occur through various mechanisms (e.g., drug absorption, metabolism, effectiveness, chemical changes). Few selected possible aspects are summarized below, with the overall aim to raising awareness and incentivize future research on the possible pharmacological interactions between food processing and drugs and their health ramifications.

A recent review on the health-promoting features of food bioactive compounds including polyphenols, carotenoids, vitamins, glucosinolates, triterpenes, phytosterols, alkaloids, capsaicinoids, polysaccharides, polyunsaturated fatty acids and bioactive peptides, concluded that there are several factors that may affect the content and bioavailability of these compounds. Indeed, one of the factors that has a significant influence on bioactive compounds is the effect of food processing (5). Some selected types of food processing and their possible interaction with drugs are listed below.

## Thermal processing

Among the traditional technologies of food processing, such as drying, addition of preservatives like salt, heating is certainly the most common one. Among many types of thermal processing deep fat frying, grilling or barbecuing are utilized both for home cooking, food service and industrial manufacturing. This unique unit operation requires high heat and the product undergoes texture changes, while a considerable amount of oil is often absorbed.

### Reduction of the enteric absorption of drugs

Fried foods, typically prepared with high-fat cooking methods, can delay gastric emptying and influence the enteric absorption of certain drugs, particularly those that are lipophilic. Traditional fried food is made by immersing it in hot oil at a typical temperature of 150–200°C. Worldwide fast-food companies supply fried foods, such as French fries, fried chicken, fried pork chops, fried sweet potatoes, fried banana chips, to count only a few. Fried foods are widely popular due to their unique taste and flavor, golden color, crisp texture and other for the consumers appealing attributes. However, some fried foods absorb a large amount of oil during the traditional frying process, and the final oil uptake can reach in extreme cases up to 50% of the total weight (6). In many fried foods, oil uptake is above 20% of the total weight. For instance, in case of potato chips, it is 34.6%, corn chips, 33.4%, tortilla chips, 26.2%, doughnuts, 22.9% of the total weight (7).

Reduction of oil uptake is possible by applying a plethora of processing techniques. The frying process, product characteristics and oil quality are key factors affecting oil absorption. At higher frying temperatures, oil absorption is usually reduced, as the process is shorter and the enhanced crust formation acts as a physical barrier for oil imbibition. For instance, potato crisps fried at 120°C have higher oil content compared with their counterparts fried at 180°C (7). Various innovative frying processes have been developed to reduce oil uptake, like vacuum frying (VF), microwave frying (MF), microwave-assisted vacuum frying (MVF), ultrasound combined microwave vacuum frying (UMVF), air frying, and radiant frying (8). Baking is also utilized and could reduce oil uptake significantly (9).

Notably, in addition to oil uptake, frying produces volatile/non-volatile compounds which darken the food’s color, generate aromas, and develop unique crust and textures (10, 11).

The high oil content of the fried foods could interact with the drug enteric coating. Some medications have enteric coatings to protect them from stomach acid and improve absorption in the intestines (e.g., erythromycin, pancrelipase, proton pump inhibitors, budesonide). Hence, different processing methods of the same food product, such as deep fat frying or baking may considerably and differently affect the dissolution of enteric coatings, by this way potentially altering the drug’s release profile.

### Generation of pleiotropic and harmful aldehydes

To properly and comprehensively analyze the frequent interference of food frying with the enteric absorption of various medicines, one must take into account also that the high temperature of the oil may induce harmful chemicals, above all it enhances the oxidation of oil lipids, of course via non enzymatic pathways, and it also induces the formation of several other potentially harmful compounds, like acrylamide (due to a Maillard reaction), polycyclic aromatic hydrocarbons and heterocyclic amines. Several strategies to reduce acrylamide formation during food processing (e.g., frying, baking) were recently reviewed (12, 13).

Extensively studied since many decades is the oxidation of polyunsaturated fatty acids (PUFAs) at the level of the carbon–carbon double bond(s), a free radical chain reaction called lipid peroxidation, which is leading to alkoxy-, peroxy-, lipid radicals and lipid hydroperoxides; the latter molecules are highly unstable, so easily break down generating various still reactive but much more diffusible end-products, of which the most studied are aldehydes, since provided with different toxicological properties (14–16).

Of the several aldehydes stemming from PUFAs, the quantitatively and qualitatively more important are malonaldehyde, generated from both ω-3 and ω-6 PUFAs, 4-hydroxyhexenal (HHE) from ω-3 PUFAs and 4-hydroxynonenal (HNE) from ω-6 PUFAs (16). These carbonyl compounds, in particular HHE and HNE, readily form stable adducts with proteins and lipids by this way deranging their structure and function, for example at the level of the intestinal epithelial layer, and also exerting strong pro-inflammatory stimuli (16, 17).

Indeed, the generation of these toxic aldehydes by high fat frying has been shown for four different vegetable oils, all containing both ω-3 (linolenic acid) and ω-6 (linoleic acid) PUFAs namely soybean oil, sunflower oil, rapeseed oil and corn oil (18). In the food cooked with any of these four vegetable oils the production of relevant amounts of HNE was demonstrated (19). Previously, the same harmful aldehyde was consistently detected in relatively high concentration in French fried potatoes obtained from six different fast food restaurants (20). HNE is most likely the most toxic aldehyde produced during lipid peroxidation (16), a biochemical process which in the thermal processing of food is driven and sustained by the reactive oxygen species generated in high amount by heat (21).

As regards HNE, a very important observation was recently made that points to an additional mechanism of interference by this aldehyde with the pharmacological effect of orally taken drugs. In fact, in a screening study actually simulating a clinical worst case scenario, bags for endogenous saline infusion, catheters and disposable syringes, all made with plastic polymers, were shown to leach HNE in their medicinal contents (22). Based on the recognized readiness of this reactive and diffusible carbonyl to form protein and lipid adducts, a direct addition of HNE and maybe other reactive aldehydes to active pharmacological principles (API) appears a quite likely event.

Indeed, deep fat fried food by-products are at present considered as a significant dietary contributor to the most common chronic diseases especially affecting the elderly, including atherosclerosis, cancer and hypertension (23). Hence, a spectrum of alternative types of food processing should be taken into consideration (e.g., baking, air frying).

### Methylglyoxal, glyoxal and AGEs, as potentially dangerous products of Maillard reaction

Methylglyoxal (MG) and glyoxal (G) are two alpha-ketoaldehydes (dicarbonyls) that may derive from autoxidation of polyunsaturated fatty acids and glucose, and are consistently present in foodstuff rich in these nutrients, in which they are mainly formed via the Maillard reaction. The two reactive dicarbonyls form adducts with proteins, by this way being potentially cytotoxic, in the case of food potentially enterotoxic, and, in addition, they readily lead to a quantitatively considerable formation of advanced glycation end products (AGEs), chemical species with strong pro-oxidant, pro-inflammatory and cytotoxic properties (24, 25). In fact, following the binding of AGEs to the membrane receptors for advanced glycation end products (RAGEs), a redox signaling is generated that activates the inflammatory reaction and induces an oxidative burst (25, 26).

An excessive production of MG and G, as it could occur especially at very high temperatures and long time exposures, would contribute to bring in the gut a mixture of reactive species, with likely derangement of enteric structure and function (24, 27). An indirect, while quite effective, mechanism of malabsorption not only of nutrients but also of medications. But, by far more important as regards food processing interaction with drug pharmacokinetics, is the demonstrated reaction of dicarbonyls and AGEs with membrane transporters like ABCA1 (ATP binding cassette sub-family A member 1), considerably expressed on the surface of the intestinal epithelial barrier. Indeed, in the gut, one of the largest groups of membrane transporters, namely the ABC family, allows the intracellular uptake of a great variety of molecules including many drugs, mainly lipophilic ones. Years ago, glyoxal, but not methylglyoxal, was shown to destabilize the activity of ABCA1, in cultured human skin fibroblasts and murine macrophages, even if the protein synthesis of the transporter was not affected (28). Much more recently, using murine macrophages, the same group demonstrated this time that the advanced glycation of human albumin (AGE-albumin) was able to markedly inhibit the synthesis of the membrane transporter ABCA1. Moreover, those authors elucidated the mechanisms of such an effect of AGE-albumin, namely a net enhancement of ABCA1 ubiquitination and consequent proteasome degradation (29). Thus, based on these findings, one should most likely expect a significant contribution also by a “dicarbonyl stress” to the potential interference of food thermal processing on the uptake of drugs.

### Thermal oxidation of sterols: oxysterols and oxyphytosterols

The high temperatures that could be reached in the industrial food processing, and during frying, grilling or barbecuing of a large variety of foodstuffs, generate another wide class of potentially harmful compounds, namely stemming from the heat-dependent oxidation of sterols, quantitatively well represented in foods both of animal and vegetable origin. By far more investigated and characterized is the family of oxysterols, 27-carbon atoms compounds derived from the oxidation of cholesterol, while the oxyphytosterols, i.e., the oxidation products of phytosterols, still need further characterization as regards their possible detrimental effects on health.

The thermal processing of food of animal origin has clearly been demonstrated to induce the non-enzymatic formation of a large variety of oxysterols (30, 31) the most dangerous of which are certainly 7β-hydroxycholesterol (7βOHC) and 7-ketocholesterol (7KC), since provided with highly cytotoxic and pro-inflammatory properties (32–35). An indirect interference with the gut absorption of medicines is that affordable by a mixture of oxysterols like that present in the gut of people fed a Western type of diet, because it contributes to damage the intestinal epithelial layer, in particular by deranging both tight and adherens junctions (36, 37). The impairment of the intercellular adhesion molecules should then mainly affect the paracellular absorption of drugs, i.e., the main mechanism of intestinal uptake of hydrophilic drugs (38).

On the other hand, hydrophobic drugs are absorbed via a transcellular route, taken up by active transporters (38), hence the high oxysterols’ intake due to thermal processed food might represent an obstacle for the physiological action of the numerous multidrug protein transporters selectively expressed along the gastro-intestinal tract (39). In principle, oxysterols could readily oxidize these transporters through the up-regulation of reactive oxygen species production, and, in addition, two of them, namely 5α,6α-epoxycholesterol and 5β,6β-epoxycholesterol, were even shown to form protein adducts by reacting with the ε-amino group of lysine (protein sterylation) (40). However, the suggested addition reaction of defined oxysterols with drug protein transporters is at present just a speculation, since no specific literature is available yet. The same lack of specific literature (addition reaction with membrane transporters) applies for HNE and other aldehydes, any way shown to readily undergo addition reaction with proteins.

In the food of vegetable origin, steroids are largely represented and are named phytosterols, compounds having a structure similar to cholesterol (41), and like cholesterol being prone to autoxidation, leading to the generation of quite a number of oxyphytosterols. As in the case of oxysterols, the quantitatively more relevant oxyphytosterols are 7 K- and 7OH- derivatives of sitosterol and campesterol, but also the epoxy derivatives may reach elevated concentrations in the diet (41, 42).

A clear example of heat-induced oxidation of phytosterols, was provided by a careful and detailed analysis of the oxyphytosterol content of refined rapeseed oil, really one of the most used edible oils worldwide, before and after thermal treatment. Two different temperatures were considered, 60°C and 180°C, and various heating times. The total oxyphytosterol content detected in the rapeseed oil heated at 180°C for 15 min resulted to be four times as higher as that quantified in the 60°C heated oil (43). Even in this study, actually in line with a much earlier report (44), 7 K- and 7OH- and epoxide derivatives of sitosterol and campesterol represented the major oxyphytosterols detected (45).

While systematic analysis and characterization of these compounds are still missing, oxyphytosterols were already recognized to exert cytotoxicity when present in relatively high amounts in the diet (42, 45). Consequently, they in principle could affect the enteric absorption of those medicines that are taken up by the intestinal epithelial layer, if some cells of this layer are irreversibly damaged by phytosterol oxides. Further, one should expect that oxyphytosterol-epoxides, like oxysterol-epoxides could lead to stable sterylation of proteins, possibly including multidrug transporters. Indeed, protein sterylation by oxysterols and oxyphytosterols appears to be a very interesting emerging issue that deserves to be elucidated soon.

As regards the possible toxicity of oxysterols and oxyphytosterols stemming from thermal processing of food, the actual extent of it might be modulated by the adopted diet. For instance, many representative compounds of the Mediterranean diet, like tocopherols, fatty acids, polyphenols, argan and olive oils, different cytotoxic effects of 7βOHC and 7KC result to be quenched (46, 47).

### Heat-induced production of recognized cytochrome P450 isoenzymes inducers: acrylamide, heterocyclic amines and polycyclic aromatic hydrocarbons

Not only deep-fat frying, but also, while in a relatively less amount, air frying, barbecuing and grilling give rise to other harmful compounds, in particular acrylamide, polyciclic hydrocarbons, namely benzo[a]pyrene, benzo[a]anthracene, benzo[b]fluoranthene, chrysene, and heterocyclic amines (48, 49). Notably, all these compounds are metabolized by various hemoproteins of the cytochrome P450 (CYP) superfamily, and induce their own metabolism, as they are potent inducers of CYP enzymes (50, 51). Since several components of the CYP superfamily are involved in the metabolism and biotransformation of a great number of drugs, a possible interference by the aforementioned classes of side-products of thermal processing of food appears very likely, even if specific proofs are not available yet in the literature.

**Figure 1:**
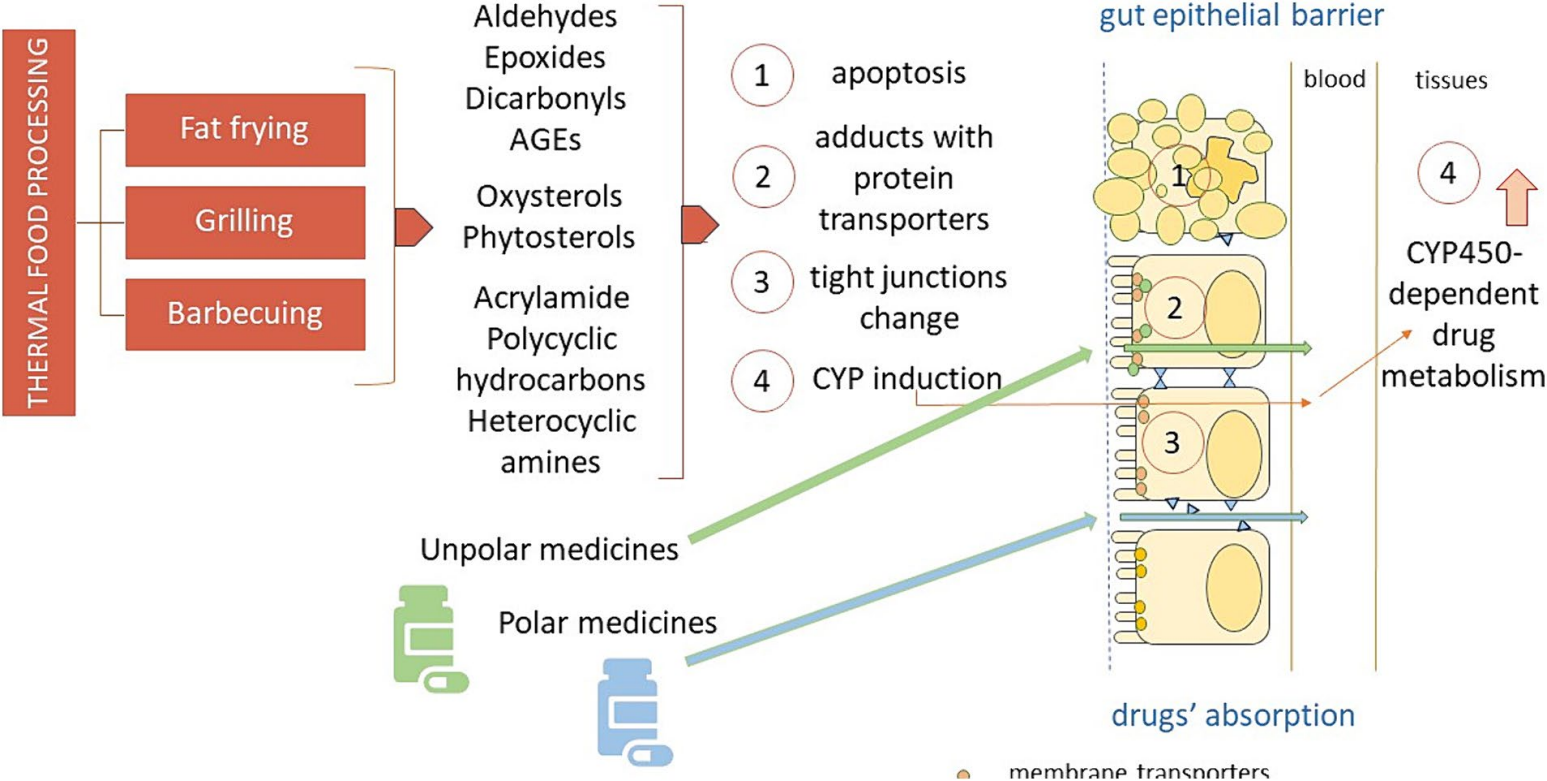
The different mechanisms by which thermal processing of food may interact and affect the pharmaceutical action of orally administered drugs. AGEs: advanced glycation end-products; CYP450: cytochrome P450.

Figure 1 depicts the various mechanisms by which thermal processing of food may interact and affect the pharmaceutical action of several drugs, that is interfering with their intestinal uptake, both directly, by inhibition of or competition with the gut multidrug transporters and indirectly, by damaging the epithelial cells and the intercellular junction molecules of the intestinal barrier, and by affecting drug metabolism.

The heat-mediated oxidation of food may produce elevated amounts of electrophilic compounds, such as aldehydes and epoxides, ready to make irreversible adducts with nucleophilic, i.e., polar drugs, like some antibiotics and antiviral active compounds, by this way most likely altering their pharmacokinetics. On the other hand, the intestinal absorption of the numerous electrophilic drugs is mainly regulated by uptake transporters and export proteins expressed on the plasma membranes of the gut epithelial lining (52), susceptible to the attack by aldehydes and epoxides stemming from heat processed food. Not least of all, several harmful byproducts that could arise from heated food may quench the pharmacological effects of drugs metabolized by CYP enzymes.

## Thermal processing of medicinal herbs

Thermal processing (e.g., boiling, drying, heating, steaming) are frequently reported during the preparation of various traditional herbal medicines (53). Before their medical use, thermal processing methods are frequently utilized to treat the traditional Chinese medicine (TCM) and herbal materials to improve their efficacy and/or reduce their side effects. In some cases, the thermal processing seems to alter the herbal chemical components (53).

Understanding the temperature-dependent chemical reactions of herbal materials is necessary to explore the underlying mechanisms and optimize the procedures of thermal processing. Numerous cases have been reported on how thermal processing could influence the phytochemical contents, and sometimes could alter the bioactivities of these plant-derived foods. In some cases, the formation of new phytochemical conjugates was occurring.

Thermal-induced molecular conjugates are utilized for their antioxidant, anti-cancer, anti-diabetic, and anti- inflammatory activities. It is believed that the energy derived from such thermal processing helps to overcome the energy barriers and hence facilitates the phytochemical transformations. Frequently, the observed thermal-induced change in phytochemical contents could be attributed to the Maillard reaction. However, in other cases, chemical reactions such as degradation, transglycosylation, deglycosylation, dehydration, and oxidation were proposed. Yet, the reaction mechanism remains to be elucidated (53).

*A typical example is* Fritillaria *thunbergii* Miq. (*F. thunbergii*, family Liliaceae), the underground bulbs officially listed in the Chinese Pharmacopoeia as “Zhebeimu,” that has been considered an important antitussive, expectorant, and antihypertensive agent in TCM for thousands of years. The major active components of the *F. thunbergii* are steroidal alkaloids, including peimine, peiminine, zhebeinine, zheberine, and zhebeinone, among others (54, 55). Sun-drying method, when compared with microwave drying, yielded significantly lower peimine and peiminine total content, protein, soluble amylose, resistant starch (RS), solubility, swelling power, and relative crystallinity, while increased the insoluble amylose content and the water-binding capacity. Microwave-dried sample showed significant changes in starch content. Low levels of rapidly digestible starch and glucose and high RS levels were found in the hot air-dried and freeze-dried samples. It was concluded that *F. thunbergii* flour can be used as medicinal excipient and health product, especially when subjected to chemical or physical treatment (50).

Another example of a popular TCM is *Polygonum multiflorum* (Heshouwu), used for rejuvenate purposes. Steaming involved in the processing of *P. multiflorum* root extract leads to a new chromatographic peak in HPLC analysis. Its intensity increased with steaming times ranging from 8 to 48 h (53). It was shown that 2,3-dihydro-3,5-dihydroxy-6- methyl-4(H)-pyran-4-one and 5-hydroxymethyl furfural were confirmed (56). Likewise, in a subsequent study using steamed *P. multiflorum* extract, eleven new compounds were identified (4-furanones, 2-furans, 2-nitrogen compounds, 1-pyran, 1-alcohol and 1-sulphur compound) (57). Among these 11 newly formed products following steaming, 5- hydroxymethyl-furfural was further tested and demonstrated to have potent radical scavenging properties (57). In both studies, the Maillard reaction was believed to be involved in the formation of these new conjugates.

*Panax ginseng* is a further traditional medicinal herb treasured as health promoting tonic. Traditionally, *P. ginseng* could be processed into white ginseng (sun-dried ginseng) or red ginseng (steaming fresh ginseng at 95–100°C for a certain time duration), depending on the presence of this steaming step (53, 58). Previously, numerous studies reported on *P. ginseng* rich phytochemical contents (53, 59, 60). Panaxydol, a member of the class of polyacetylenes, is a phytochemical isolated from the root of *P. ginseng*. The steamed red ginseng was reported to contain higher panaxydol amount, compared to the un-steamed white ginseng sample (58). This finding bears medicinal significance, as this small molecule was shown to exert a net apoptotic effect in an anticancer study (61). One suggested possible route for the formation of panaxydol is via the oxidation of panaxytriol, another polyacetylene, following the thermal processing (53, 58).

In TCM, it is common to include the use of herb pair or herbal formula for treating diseases. Two or more different medicinal herbs could be combined in different ratios and processed into a single decoction (53, 62). It is believed that the combined herbs in an herbal formula could work synergistically and enhance the medicinal values. During the preparation of decoction from herbal formula, heating and boiling are usually involved. Some of these new phytochemical conjugates derived from herbal formula were not detected in any of the single herb (53). More recently it was shown that the primary structure of some ginsenosides can be modified to produce secondary ginsenosides through natural microbiota, or by various processes during the preparation of the phytotherapeutic product, such as heating or drying. Different species of genus *Panax* contain numerous compounds with diverse and important biological properties such as immunomodulatory, anti-inflammatory, and anti-cancer properties (63).

## Nonthermal processing

Nonthermal processing refers to minimally processed food techniques to preserve foods without the use of high heat, allowing processors to improve quality, nutritional aspects, efficiency and superior consumer products. Some nonthermal processing include: 1. High-pressure processing (HPP), which is a non-thermal food and beverage preservation method that guarantees food and drink safety and achieves an increased shelf life, while maintaining the high organoleptic and nutritional attributes of fresh products. 2. High-pressure homogenization (HPH), which is based on the same principles as homogenization process used in the dairy industry to reduce the size of fat globules, but it works at much higher pressures (100–400 MPa). 3. Pulsed electric fields (PEF) processing, which applies high voltage pulses (20–80 kV/cm) with a duration of milliseconds to microseconds to treat foods placed between two electrodes. For solid foods, due to the large gap required between the electrodes of the treatment chamber, and the power limit of the pulse generator, typical lower voltage pulses are applied (1–8 kV/cm).

The aforementioned nonthermal processing technologies offer what could be defined as “cold pasteurization” (64), a quite advanced application for PEF and HPP. Other processing technologies such as ozone and hydrogen peroxide treatments, are used in limited cases such as sanitization of dairy supply chain (65) and water (66). Gamma irradiation is yet another non-thermal process, but, due to consumer issues, its utilization is very limited (e.g., for spices) (67). Plasma technology is a further very promising minimally food processing technology. The use of plasma offers favorable potential owing to its different attributes including non-thermal food processing, enzyme inactivation, removal of pesticides toxin, less damage to food, low nutritional losses, and high quality of the final products (68). This technology is still under development and could become a very promising non thermal alternative in the near future.

### High-pressure processing

A typical example of the effect of nonthermal minimally processed food is highlighted when comparing the *in vitro* bioavailability of isoflavones from soymilk-based beverages, high-pressure processing (HPP) (400–600 MPa, holding times from 1.5 to 6 min), pulsed electric field (PEF) (35 kV cm^−1^) and typical regular thermal treatment (TT) (90°C for 1 min). The isoflavones concentration was found to be higher in HPP-treated samples (38.5%) whereas, in TT and PEF products, the range was significantly lower (25–26%) (69). These data highlight that processing plays a pivotal role in the bioavailability of isoflavones as well as many other active compounds.

It is known that flavonoids contained in grapefruit juice, such as naringinin and hesperidin, are responsible for the inhibition of transmembrane transporters, which play a role in the passage of several drugs from the intestinal lumen within the bloodstream (3). Thus, it clearly indicates that processing could have a significant potential interaction with drugs. As HPP orange and grapefruit juice are becoming quite popular currently, consumers are exposed to various levels of active compounds, such as relatively high concentrations of certain flavonoids. For instance, it was reported that the coadministration of drugs such as acebutolol, celiprolol or fexofenadine with grapefruit juice, or atenolol, ciprofloxacin, and fexofenadine with orange juice, decrease the oral bioavailability of antihypertensive, antibiotic and anti-histaminergic drugs (3, 70). In particular, the grapefruit juice can block the action of intestinal CYP3A4, the amount of which varies from person to person in the small intestine, so grapefruit juice may affect people differently even when they take the same drug. Besides the possible interference with drug metabolism, grapefruit juice can affect drug transporters proteins and the final result is that less drug enters the blood.

Besides grapefruit juice, *Ginkgo biloba*, an important herbal compound and a dietary supplement, has actually been proven to interfere with the effectiveness of some medications (71). *Ginkgo biloba* is used to improve brain performance and reduce fatigue (3). It is also commonly utilized by people experiencing cognitive decline; healthy adults seeking to improve performance or prevent a decline; and elite performers seeking to optimize their cognitive performance (72). The substances contained in the *Ginkgo biloba* that have pharmacological properties are flavonoids and triterpene lactones (gingkoloids and bilobalids). As a result, *Gingko biloba* is able to reduce platelet aggregation, and acts on the CPY2C9 and CYP3A4 isoenzymes, inhibiting the microsomal metabolism of warfarin (3). Therefore, herbal preparations containing *Ginkgo biloba* should be avoided in patients treated with antiplatelet or anticoagulant drugs (73). It should be also noted that products that are HPP-treated and contain ginkgo seed protein (GSP) showed markedly improved heat stability and emulsifying properties compared to the untreated GSP (74) and consequently may interfere with the effectiveness of some medications as aforementioned.

### High-pressure homogenization and microfluidization

Another common nonthermal processing is high-pressure homogenization (HPH), that combines, in addition to high pressure action, some other physical effects (e.g., cavitation, shear stress, turbulence). Like HPH, microfluidization (MF) is a method used for production of micro and nanoscale size materials. It is commonly used both in pharmaceutical and food industry to make liposomal products, emulsion and to produce dairy products. Both processes could affect bioavailability of some active food compounds. For instance, HPH and MF processes were utilized in the emulsification of krill oil. Emulsions produced through MF exhibited several noteworthy advantages over those generated by HPH. Most prominently, MF-prepared emulsions featured smaller and more uniformly distributed particles, in stark contrast to the less uniform particles generated by HPH. Moreover, MF-based emulsions demonstrated significantly enhanced oxidative stability during storage. Astaxanthin degradation occurred at a substantially lower rate (38.1 and 89.4% for HPH and MF, respectively) (75). In *in vitro* simulated digestion, MF formulations exhibited superior stability and markedly higher bioaccessibility of food active components in comparison to their HPH counterparts. Significant increase in the release of free fatty acids was observed during the intestinal phase of digestion in MF emulsions, indicating an improved lipid digestion process (75). These findings highlight significant differences for ω-3 fatty acids, that can interact with blood thinning drugs and could have possible adverse effects, or determine therapeutic failure.

### Pulsed electric fields

PEF deserves a closer look due to its unique capabilities as highlighted in the study of clinical applications and immunological aspects of electroporation-based therapies, food and medicine (76, 77). Consequently, PEF treatment is considered to be a promising technology that has in the last years received considerable attention in food and biotechnology related applications (78).

PEF impact causes membrane permeabilization, a process termed as electroporation (EP), and leads to an increased permeability of the membrane to ions and molecules (79). Depending on the intensity of the treatment applied (e.g., external electric field, single pulse duration, treatment time) and the cell characteristics (e.g., size, shape, orientation in the electric field), the viability of the electroporated cell can be preserved by recovering the membrane integrity (reversable permeability, REP). Conversely, EP can permanently lead to cell death (irreversible permeability, IRP). REP is a procedure commonly used in molecular biology and clinical biotechnological applications *in vivo* to gain access to the cell cytoplasm in order to introduce or deliver drugs (e.g., oligonucleotides, antibodies, plasmids) (78, 80). Most of food and biotechnology related applications of PEF are based on irreversible permeabilization of the cell membranes and mainly include “cold” pasteurization of liquid foods and disinfection of wastewater by means of microbial inactivation (78).

Typical applications of PEF technology in food processing include extended shelf life (81): 1. Apple juice; 2. Orange juice; 3. Milk; 4. Liquid egg processing (in combination with adopting a hurdle strategy); 5. Processing of green pea soup. Other applications include: Beer (inactivation and sublethal impairment of *Lactobacillus plantarum* a microorganism that can spoil beer). Most recent utilization of PEF includes (82): 1, Pineapple juice and coconut milk mixture; 2. Red wine; 3. Milk; and 4. Human milk (i.e., processing conditions were optimized to reduce bacterial counts in donor human milk, and to evaluate its effect on the bioactive proteins). Extraction of bioactive compounds in food using PEF processing is also a very common approach.

Nanosecond PEF (nsPEF) processed milk retaining over 60% of lysozyme, lactoperoxidase and lactoferrin, and 100% retention of xanthine oxidase and immunoglobulin A was reported. Additionally, the loss of milk proteins was smaller for samples treated with nsPEF (e.g., high voltage, high intensity pulses are used, with durations of 10–300 ns and electric fields of ~10 kV/cm to 300 kV/cm) in comparison with typical regular pasteurization process. These data indicated that nsPEF is a promising novel pasteurization method (83). However, the application of PEF to solid foods is much more difficult, mainly because microbial inactivation in this case is relatively unrealistic (82).

Despite many scientific studies on the principles and applications of PEF technology published to date, and the fact that PEF was introduced into the food industry many years ago, this technology is still considered as an emerging one. In the European Union there is no special legislation on food processed with PEF. In general, the use of this technique is coordinated by the Novel Food Regulation (EU) 2015/2283,^1^ but implementation of PEF into production does not automatically mean that the food becomes “novel” (84). Novel food acknowledgement should be closely considered and evaluated when a new technology is to be implemented.

Although electroporation (EP) and electropermeabilization (EPP) are frequently used as synonyms, EP is related strictly to the aqueous pores formed in the lipid membranes of the cells, while EPP is related to all the events involved in the membrane permeabilization process, including modulation of membrane channels, cellular biophysical and biochemical changes (77). In medicine, EP is a platform technology for drug and gene delivery. When applied to cell *in vitro* or tissues *in vivo*, it leads to an increase in membrane permeability for molecules which otherwise cannot enter the cell (e.g., siRNA, plasmid DNA, and some chemotherapeutic drugs) (85). In oncology, reversible electropermeabilization (REPP) is applied for the intracellular transport of chemotherapeutic drugs as well as the delivery of genetic material in gene therapies and vaccinations. The physical changes of the membrane and the immunological aspects involved in electrochemotherapy and gene electrotransfer, were recently reviewed for two important EP-based cancer therapies in human and veterinary oncology (77). The two widely used chemotherapeutic drugs are bleomycin, a chemotherapy agent employed to treat various malignancies, including head and neck malignancy, lymphoma, and testicular tumors, among others,^2^ and cisplatin, a chemotherapy drug used to treat testicular, ovarian, bladder, head and neck, lung and cervical cancer, among others.^3^

## Possible increased absorption of nutrients related to non-communicable diseases due to food processing

**Figure 2:**
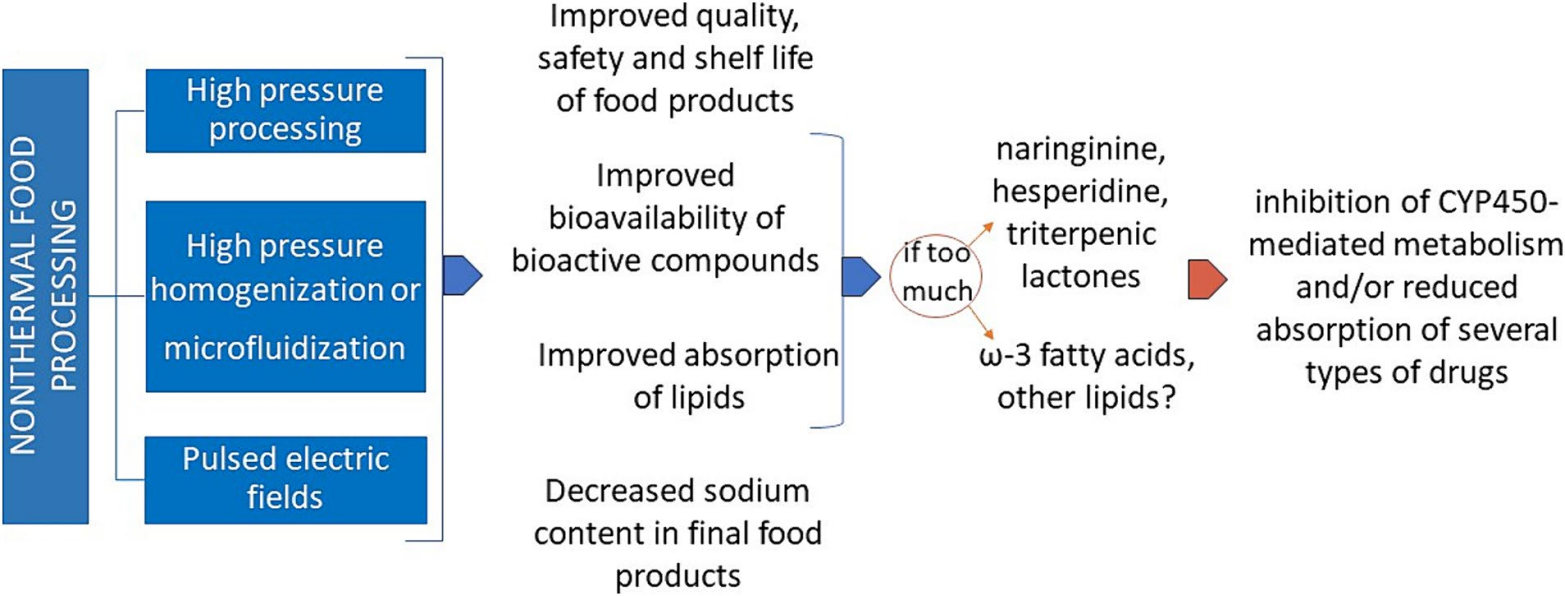
Various advantages and some possible drawbacks of non-thermal food processing. CYP450: cytochrome P450.

As schematically reported in Figure 2, non-thermal processing often allows a better bioaccessibility and bioavailability of the active components present in the so processed foodstuff. Even if this fact appears positive *per se*, maximal attention must be given to the possible interference of an excessive intake of certain ions and biochemical compounds in the people under pharmacological treatment for non-communicable diseases (NCDs), mainly cardiovascular, respiratory, gastrointestinal, obesity, type-2 diabetes and other metabolic diseases. Typical examples are the deleterious interfering effects of a potential excessive uptake of potassium and phosphorus on the drug treatment of hyperkalemia and hyperphosphatemia, two main metabolic alterations in chronic kidney insufficiency (86), or that of sodium during treatment of the high blood pressure (87).

NCDs were connected to what is known as ultra processed foods (UPFs) as described by the NOVA classification system (88). UPFs is based on food processing classification. In essence, the classification is rather based on formulation focusing mainly on added fat, carbohydrates and other processing ingredients such as flavor. The topic is very controversial among experts (89, 90) and will not be discussed herewith. Nevertheless, in the public domain UPFs are perceived as hazardous (91).

Of note, the possible hindrance of a correct pharmacological treatment of NCDs by UPFs, due to an enhanced bioaccessibility of defined active principles, is dependent on the alteration of the correct metabolic contest necessary, e.g., for hypertensive drugs to exert their pharmacological action, with the consequent risk of drug’s lower effect and possibly consequent overdosing with related complications. A scenario far from being uncommon, since NCDs incidence significantly increase from +55 years adults onwards, with the most occurrence and prevalence in elderly.

The Pan-American Health Organization Nutrient Profile Model (PAHO NPM) identified a variety of processed and ultra processed foods with a critical content of nutrients pathogenetically correlated with NCDs, including saturated fat, total fats, and sodium, having as a reference their recommended amounts by World Health Organization (92). It came out, for instance, that the majority of Australian people eat daily from three to five processed or UPFs containing those critical nutrients (93). In a large cohort of adults from Northern Italy, namely Emilia Romagna, renowned for ham and parmesan cheese, the mean sodium intake has been found as significantly higher than the recommended one, while that of potassium resulted to be just slightly lower to the recommended intake (94).

One should consider the fundamental physiological role of sodium and potassium. Age-related increases in blood pressure are virtually absent in populations in which individual consumption of sodium chloride is less than 50 mmol (mEq) per day. Hypertension is observed mainly in populations in which people consume more than 100 mmol (mEq) of sodium chloride per day (95). Although individual sodium intake in most populations throughout the world exceeds 100 mmol (mEq) per day, most people remain normotensive. It appears, then, that sodium intake that exceeds 50 to 100 mmol (mEq) per day is necessary but not sufficient for the development of primary hypertension.

In the Dietary Approaches to Stop Hypertension (DASH) sodium study, a reduction in sodium intake caused stepwise decreases in blood pressure. Isolated populations that eat natural foods have an individual potassium intake that exceeds 150 mmol (mEq) per day and a sodium intake of only 20 to 40 mmol (mEq) per day (the ratio of dietary potassium to sodium is >3 and usually closer to 10) (96). By contrast, people in industrialized nations eat many processed foods and thereby ingest 30 to 70 mmol (mEq) of potassium per day and as much as 100 to 400 mmol (mEq) of sodium per day (the usual dietary potassium/sodium ratio is <0.4) (97).

Food processing drastically changes the cationic content of natural foods, increasing sodium and decreasing potassium. Only approximately 12% of dietary sodium chloride originates naturally in foods, whereas approximately 80% is the result of food processing, the remainder being discretionary (added during cooking or at the table) (98).

The results and conclusions of this rather old while still highly cited report, that was pointing to a certainly minor contribution of the optional salt (NaCl) added by the consumer to the total sodium content of foodstuffs, were confirmed by a quite recent study, the size and features of which were more comprehensive (99). These authors recruited 450 adults from three different US regions, 50/50% M/F, of four races, and estimated, through already standardized procedures, the amount of sodium from the various sources, i.e., that inherent to food or deriving from food processing or added at home. The findings obtained were clearly consistent with those reported previously (98), a study was carried out only on white, mainly female, adults, from a single US region. In fact, the sodium deriving from processed food resulted to be 71% of the total, of course with some significant variations among the examined groups, being the sodium inherent to food 14%, the remaining percent amount added at home (99).

Apart from educating the public, an agreement by the food industry to limit the deviation of sodium content of processed foods from their natural counterparts appears essential. Indeed, an excessive intake of sodium with food would favor an increase of blood pressure, certainly not recommendable for +55 people. Even more important, such an overload could considerably interfere with the clinically expected effect of a given anti-hypertensive medication, a correct dosing of which could become complicated.

Besides the possible hindrance of a high sodium diet to a correct dosing of a given medication, proofs are available that support a direct alteration of the pharmacokinetics (PK) of different cardiovascular drugs. In fact, a marked impairment of the PK of the commonest antiarrhythmic drug quinidine when administered orally versus that administered intravenously was observed in healthy adult volunteers of both sexes fed a high-salt (400 mEq/day) for ten days then receiving 600 mg of the drug (100). No changes in the PK of the drug administered i.v. The reported marked drop in the bioavailability of quinidine given *per os* was occuring early after its oral administration, suggesting a pre-hepatic, most likely intestinal, impairment of the drug’s absorption operated by the dietary salt excess (100). The significantly decrease in bioavailability of quinidine, when orally given, as induced by a high dietary salt concentration was considered as mainly due to an altered metabolism or a deranged transport of the antiarrhithmic drug (or both) at the intestinal level (101).

Further studies by the same group found a similar lowering effect of high dietary salt on the bioavailabilty of verapamil, another common antiarrhithmic and antihypertensive medication. Once again, the interaction between a high sodium dietary intake and the absorption of verapamil appeared to take place at the level of the epithelial intestinal layer, most likely involving the CYP3A-dependent drug metabolism and/or the intraepithelial uptake of the drug through the action of ATP-binding cassette sub-family B member 1 (ABCB1) (102).

Another more recent investigation was performed on male adults under a dietary sodium excess, to whom a single oral dose of an angiotensin-converting enzyme inhibitor (ramipril) or one of two different angiotensin II receptor blockers (candesartan cilexetil and valsartan) or the β1-adrenergic blocker atenolol was administered. A net impairment of the PK of candesartan and atenolol was observed in the healthy volunteers fed a high sodium diet, as to that occurring in volunteers under a low sodium diet who received the same oral dose of such drugs. Conversely, the PK of valsartan and ramipril remained unchanged (103). The authors reached the same conclusion drawn by Darbar and colleagues several years before, i.e., the high sodium dietary regimen may likely affect the intestinal absorption and metabolism of various medications.

Even if a conclusive mechanistic elucidation of the interaction between high sodium in the diet and the absorption and metabolism of the above reported drugs has not been obtained yet, it certainly develops at a pre-hepatic level (first-pass drug metabolism) and it most likely involves changes of the sympatetic gut function, beyond CYP-dependent metabolism and membrane transporters (103).

From a culinary standpoint, salt boasts numerous desirable qualities that enhance the positive sensory attributes of foods and plays also a role in ensuring product safety. However, escalating worries about the adverse effects of excessive sodium chloride (NaCl) consumption have propelled research into reducing NaCl content while maintaining quality and safety. Various cutting-edge technologies for NaCl reduction (e.g., others chloride salts, non-chloride salts, vacuum curing, ultrasound drying, microwave vacuum drying, infrared radiation drying, flavor enhancers) have been explored in meat curing (104). One promising approach involves replacing NaCl with up to 70% potassium chloride salt (KCl).

In an attempt to encourage this shift, the Food and Drug Administration (FDA) has issued an advisory document, advocating for the incorporation of potassium chloride as an alternative to NaCl in food.^4^

It is essential to note that this transition from NaCl to KCl may introduce potential deleterious effects, particularly concerning an excessive uptake of potassium. This may pose challenges to drug treatments for hyperkalemia and hyperphosphatemia, two prevalent metabolic alterations in chronic kidney insufficiency (86). This calls for better communication between food professionals and physicians. Additionally, the aforementioned concerns about possible salt-drug interference provide a supplementary impetus for the exploration and expansion of food processing methods capable of delivering salt-reduced products without the additional of other salts that may have a negative impact. The subsequent section reviews selected examples of salt-reducing food processing techniques in light of these considerations.

## Positive effects of food processing on drug delivery, stability and health safety

Delivery of drugs due to their inherent attributes (e.g., sensitivity to heat and oxygen, hydrophobicity, bitter after-taste, poor dissolubility, low bioavailability, instability to gastric conditions, possible adverse effects in the gastrointestinal tract, toxicity) is quite challenging.

To overcome some of these obstacles, encapsulation, nanoparticles and other food processing were utilized (105, 106). Few examples are listed below.

### Polymers-mediated drug delivery

Delivery of therapeutics using synthetic polymers is challenging due to toxicity, immunogenicity and impaired bioavailability following administration of the latter compounds. However, natural polymers are being explored as safe for their use as a substitute for synthetic polymers. Derivatization of starches has the potential to achieve desired properties (e.g., improved solubility, stability, bioavailability) of an incorporated drug and lower-down induced toxicities. Starch structure and chemical modification methods integrating aspects of its use in developing drug delivery devices like tablets, hydrogel, and patches were described and may be applied as a reference for future chemically modified starch as excipient in drug carrier studies (107).

### Carotenoids’ bioavailability

The consumption of specific carotenoids has been associated with reduced risks of contracting a number of chronic conditions. Extrinsic factors affecting carotenoid bioavailability include food-based factors, such as co-consumed lipid, food processing, and molecular structure, as well as environmental factors, such as interactions with prescription drugs, smoking, or alcohol consumption (108). Carotenoid bioavailability varies with different food cooking and processing procedures as well as with the amounts of dietary fat, fiber, and competing compounds present in the meal (109). Upon ingestion, carotenoids are released from the food matrix and are emulsified with fat and incorporated into lipid micelles in the small intestine for absorption by intestinal enterocytes. Once thought to be taken up strictly via passive diffusion, carotenoid absorption is facilitated via membrane proteins (109). Cooking, heating, or mechanical or enzymatic processing may also increase carotenoids accessibility by softening the tissue matrix (110). Another example is pasteurization shown to increase bioavailability of processed juice compared with its fresh counterpart (111).

### Nanoparticles – biomimetics

Nanoparticles have unique biological properties which can be used for detection, prevention, and treatment of diseases, such as cancer, pulmonary diseases, etc. and for drug delivery and gene therapy as well (112–114). Nanomaterials can be created in one (nanoscale), two (nanowires and nanotubes), or three dimensions (nanoparticles) (115). Nanoparticles are a particular type of nanomaterial that can occur naturally, be created using unintentionally, or indeed be engineered on purpose (112). Numerous processing technique could be utilized to produce typical nanoparticles that could include liposomes polymer-drug conjugates, etc. To date, the utilization of food processing for the creation of nanoparticles faces some key issues and hurdles such as the need to use economical processing techniques to make edible delivery systems while also ensuring that they are safe and palatable for human consumption (116).

Whilst nanotechnology applications have been planned for a variety of benefits in the agri/food/feed chain, the use of materials that contain nanoscale particles has also raised concerns over their potential adverse effects on consumers’ health (117). Therefore, safety is of primary concern and should be carefully considered before nanoparticles could be used in foods.

A recent review focused the attention on the recent new interest in developing biomimetic plant foods (BPFs), considering the likely disassembly that plant food structures undergo during processing first, then during digestion in the human gastrointestinal tract (GI) (118). A deep insight into these modifications of the original plant food structure is needed in a way to properly design future nature-inspired food structures that could indeed safely contribute to health and well-being (118).

A recent and typical example of biomimetic is represented by the naringenin-loaded macrophage membrane-coated liposome-based nanoparticles. They are provided with distinct physicochemical compositions and biological attributes to improve the bioavailability of the carried drug specifically at the target site. The developed biomimetic nanoparticle (BNP) has shown good biocompatibility, stability, satisfactory particle size, pH-responsive drug (naringenin) release kinetics, and higher cellular uptake *in vitro* (119). Yet another study showed that oral supplementation of vegan collagen biomimetic has beneficial effects on human skin physiology (120). Biomimetics will most likely have a pivotal interaction with delivery of food components and drugs.

### Non-thermal processing for salt reduction: a crucial support to anti-hypertensive drug therapy

A reduced intake of sodium is often recommended for therapeutic reasons, especially in elderly, as mentioned before, but to deliver reduced-salt products through a process that would not affect their physicochemical and sensory and hedonic attributes, as it often happens with NaCl salt replacers, indeed represents a big challenge for food industry (121). In relation to this task, it is noteworthy to mention some successful results achieved through the adoption of defined procedures of non-thermal processing. For instance, the fruitful application of PEF in reducing NaCl content in the preparation of jerky beef has been very recently described. PEF treatment (0.52 kV/cm, 10 kV, 20 Hz, 20 μs) applied to jerky beef test samples allowed to diminish the product’s sodium content by 34% as to untreated identical meat samples, fully preserving the main characteristics and the overall acceptability of the controls (122).

At present, more reports are available as far as the HPP of food is concerned, experimentally shown to be both a direct salt reducing procedure and a technology able to enhance the safety of sodium reduced meat products. HPP (treatment at 150 MPa for 5 min) of pork meat before manufacturing breakfast sausages with a reduced salt concentration, was shown to allow salt lowering up to 1.5% without affecting the main quality features of the sausages themselves but actually minimizing the typical cooking loss of the non-HPP treated products (123). A marked prevention of cooking loss was observed in the production of ready-to-eat chicken breasts undergoing a partial substitution of NaCl with KCl, when HPP was applied for 5 min after tumbling (300 MPa) and for 3 min to the final product (600 MPa). Moreover, such a treatment allowed to maintain the physicochemical features and the sensory attributes of the food product and even improved its microbiological quality in comparison to identically processed but HPP untreated chicken breasts. Notably, while the affordable NaCl reduction with the standard non-HPP procedure was of about 25%, that achieved by HPP treatment was 50% (124).

A similar result was reported for a further type of food, namely cooked fish batter, in which a reduction by 25% of NaCl concentration was achieved through the application of HPP (300 MPa for 5 min at 25°C) (125). Just one more example that pointed out the possible adoption of this non-thermal processing to downsize sodium amount in meat, a food in which this chemical element is anyway essential to guarantee texture, microbiological safety and suitable shelf-life [see for a specific review (126)].

### Iodination

A clear example of food processing that in some way facilitates a clinically effective intake of drugs is represented by the iodization of salt as the best method of hypothyroidism prophylaxis. Iodine is necessary for thyroid hormone production and its deficiency causes mental retardation, short stature, goiter and an increased risk of death in childhood in developing countries (e.g., iodization of salt increased the survival and birthweight in some African countries). In developed countries iodine sufficiency is attained by iodization of salt and by the introduction of iodine in food processing (e.g., the use of iodine as a bread stabilizer). In regions where iodine deficiency is prevalent, iodine supplementation or iodized salt is often used to prevent hypothyroidism (127).

However, in areas with sufficient iodine in the diet, excessive iodine intake can also be a concern and may contribute to thyroid dysfunction. Patients who are taking drugs like levothyroxine to treat hypothyroidism, or propylthiouracil and methimazole to treat hyperthyroidism, or undertaking radioactive iodine therapy, must be warned of the potential drugs’ side effects due to the presence of iodine in foods.

## Forward looking

The food processing is currently facing a profound transformation, often referred to as the ‘fourth industrial revolution’. This evolution encompasses cutting-edge technologies such as precision fermentation, pervasive digitalization, gene editing, and molecular technologies (128), as well as artificial intelligence (AI), internet of things (IoT), sensors, big data, cloud computing., etc. Some argue that these advancements hold the potential to revolutionize food systems (129).

Recent strides in food biotechnology and precision fermentation (130), along with parallel developments in plant molecular farming focused on generating therapeutic proteins (131), underscore the remarkable progress occurring at the intersection of science, technology, and innovation. This progress is blurring traditional distinctions between food processing and drug production.

Various synthesis systems, ranging from wild-type to modified mammalian cells, plants, insects, yeast, fungi, or bacteria, exemplify the diversity of approaches (132). Consequently, it is reasonable to anticipate a future in which the boundaries between certain food processing methods and drug manufacturing become increasingly blurred. This paradigm shift is prompting the need for a unified and simultaneous approach to address the future development of both sectors. A notable quote from Hippocrates, “Let food be your medicine and medicine be your food,” reinforces the intrinsic connection between these domains, advocating for an integrative and synergistic approach. In essence, the evolving landscape suggests that the realms of food processing and drug development will likely converge, driven by shared scientific principles and technologies.

## Conclusion

The preceding data underscore the dearth of elucidation on explicit interactions between food processing and their potential beneficial or adverse drug interaction consequences, which may precipitate in some cases in health hazards. Noteworthy are the plausible alterations in herb new molecular conjugates, drug absorption and efficacy, thereby instigating health implications and possible negative ramifications. Consequently, this discernible gap necessitates intensified and targeted scientific inquiry into the myriad conceivable interactions among food composition, food processing, and pharmaceutical agents, alongside a comprehensive investigation of the underlying mechanisms at play. It would be also interesting to determine if new molecular phytochemical conjugates may possess other therapeutic potentials undiscovered yet. The nebulous nature of this field demands substantial scrutiny and collaborative efforts across diverse domains, encompassing medicine, pharmacology, nutrition, food science, food technology and food engineering.

## Author contributions

GP: Conceptualization, Writing – original draft, Writing – review & editing. EB: Supervision, Writing – review & editing. IS: Conceptualization, Writing – original draft, Writing – review & editing.

## Footnotes

^1^ https://eur-lex.europa.eu/legal-content/en/TXT/?uri=CELEX%3A32015R2283

^2^ https://go.drugbank.com/drugs/DB00290

^3^ https://www.macmillan.org.uk/cancer-information-and-support/treatments-and-drugs/cisplatin

^4^ https://www.fda.gov/food/cfsan-constituent-updates/fda-issues-draft-guidance-regarding-use-alternative-name-potassium-chloride-food-labeling
